# Light harvesting in purple bacteria does not rely on resonance fine-tuning in peripheral antenna complexes

**DOI:** 10.1007/s11120-024-01107-4

**Published:** 2024-06-21

**Authors:** Erika Keil, Heiko Lokstein, Richard Cogdell, Jürgen Hauer, Donatas Zigmantas, Erling Thyrhaug

**Affiliations:** 1 TUM School of Natural Sciences, Department of Chemistry, Technical University of Munich, Lichtenbergstrasse 4, 85748 Garching, Germany; 2 Department of Chemical Physics and Optics, Faculty of Mathematics and Physics, Charles University, Ke Karlovu 3, 121 16 Prague, Czech Republic; 3 Institute of Molecular, Cell and Systems Biology, University of Glasgow, Room 402 Davidson Building, Glasgow, G12 8QQ Scotland; 4 TUM School of Natural Sciences, Department of Chemistry and Catalysis Research Center, Technical University of Munich, Lichtenbergstrasse 4, 85748 Garching, Germany; 5 Chemical Physics, Lund University, Naturvetarvägen 16, 22362 Lund, Sweden

**Keywords:** Light-harvesting, Energy transfer, Ultrafast spectroscopy, Excitons

## Abstract

The ring-like peripheral light-harvesting complex 2 (LH2) expressed by many phototrophic purple bacteria is a popular model system in biological light-harvesting research due to its robustness, small size, and known crystal structure. Furthermore, the availability of structural variants with distinct electronic structures and optical properties has made this group of light harvesters an attractive testing ground for studies of structure–function relationships in biological systems. LH2 is one of several pigment-protein complexes for which a link between functionality and effects such as excitonic coherence and vibronic coupling has been proposed. While a direct connection has not yet been demonstrated, many such interactions are highly sensitive to resonance conditions, and a dependence of intra-complex dynamics on detailed electronic structure might be expected. To gauge the sensitivity of energy-level structure and relaxation dynamics to naturally occurring structural changes, we compare the photo-induced dynamics in two structurally distinct LH2 variants. Using polarization-controlled 2D electronic spectroscopy at cryogenic temperatures, we directly access information on dynamic and static disorder in the complexes. The simultaneous optimal spectral and temporal resolution of these experiments further allows us to characterize the ultrafast energy relaxation, including exciton transport within the complexes. Despite the variations in PPC molecular structure manifesting as clear differences in electronic structure and disorder, the energy-transport and—relaxation dynamics remain remarkably similar. This indicates that the light-harvesting functionality of purple bacteria within a single LH2 complex is highly robust to structural perturbations and likely does not rely on finely tuned electronic- or electron-vibrational resonance conditions.

**Supplementary Information:**

The online version contains supplementary material available at 10.1007/s11120-024-01107-4.

## Introduction

The peripheral light-harvesting complex 2 (LH2) is a pigment-protein complex (PPC) serving as a light-harvesting antenna in the photosynthetic apparatus of many species of purple phototrophic bacteria. It features a ring-like structure formed from 7 to 9 identical subunits—the so-called α/β subunits—each of which consists of two polypeptides binding one strongly coupled pair of bacteriochlorophyll (BChl) *a* molecules, one weakly coupled BChl, as well as one of several possible carotenoids depending on bacterial species. Although the core feature of repeating α/β subunits is conserved across all LH2 variants, there is significant variation in the subtle subunit structure, carotenoid content, and the overall size of the ring (Blankenship 2014; Law et al. 2004; Gardiner et al. 2021).

**Fig. 1:**
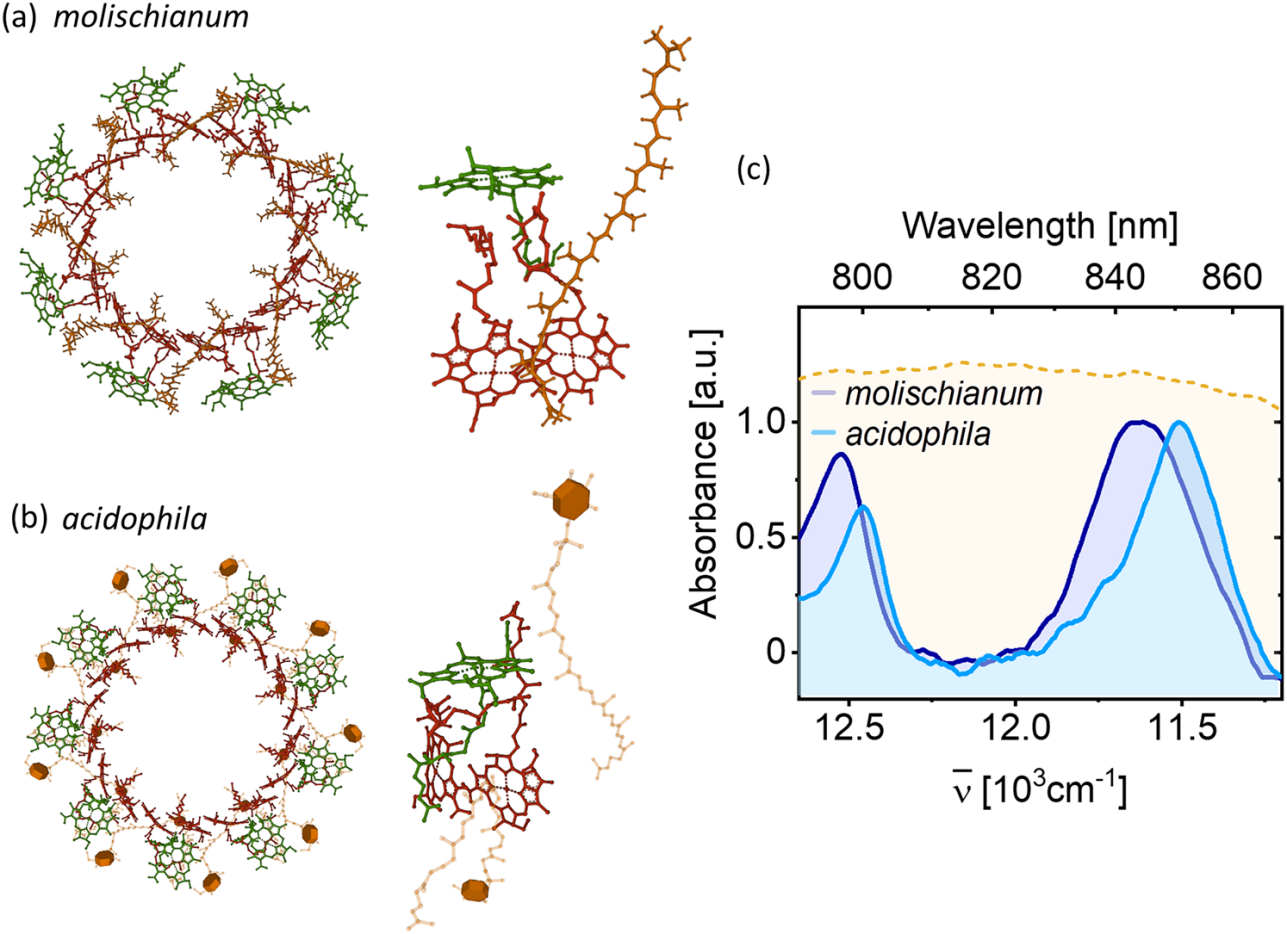
Pigment arrangement in the LH2 complexes of *Rhodospirillum (Phaeospirillum) molischianum* (**a**) and *Rhodopseudomonas acidophila* (**b**); in each case, the whole pigment complex is shown on the left, and the pigment arrangement in the respective α/β subunits is shown on the right (Koepke et al. 1996; Papiz et al. 2003). The filled feature in (**b**) represents the glucose unit of rhodopin glucoside. Both LH2 species show distinctive optical absorption features near 800 and 850 nm, as shown in (**c**). Spectra were recorded in 1:2 buffer: glycerol solution at 80 K. The yellow-filled spectrum in (**c**) is the NOPA pulse spectrum used in the femtosecond spectroscopy experiments

A common feature for all LH2 variants is the separation of the BChl pigment pool into two distinct structures: one comprising strongly coupled BChl pairs (Fig. 1, red pigments) and one of weakly coupled BChls (Fig. 1, green pigments). This separation results in the formation of two characteristic optical absorption features: a BChl-like absorption band centered at ~ 800 nm originating from the weakly coupled BChl ring and an intense, near-Gaussian band at ~ 850 nm originating from the ring of strongly coupled BChls (Fig. 1c). These absorption bands and the associated ring structures are generally referred to as the B800 and B850 band/ring, respectively (Blankenship 2014).

The light-harvesting functionality of these complexes is based on light capture and subsequent intra-ring transport in both the weak (B800) and intermediate (B850) regime of electronic coupling strength, in addition to the B800→B850 band-to-band transfer process (Thyrhaug et al. 2021). This diver*s*ity of transport processes occurring within the complex, in conjunction with known crystal structures for several commonly expressed variants, has led to the recognition of LH2 as an excellent candidate for studies of structure–function relationships in excitonic soft-matter systems. A wide variety of spectroscopic and theoretical approaches have been used in this body of work, where research efforts have been in the context of its role as a light-harvester in photosynthesis (Sundström et al. 1999; Cogdell et al. 2006) and as a model system for more general studies of soft-matter exciton dynamics. The latter include studies on vibronic coupling (Chachisvilis et al. 1997; Jang and Mennucci 2018), exciton motion and delocalization (Perlík et al. 2015; Caycedo-Soler et al. 2018), and exciton trapping (Polívka et al. 2000; Freiberg et al. 2003).

Although direct exploration of structure–function relationships by comparative studies of photoinduced dynamics in LH2 extracted from different organisms has appeared in several works (Wu et al. 1996; Kennis et al. 1997; Rondonuwu et al. 2004; Ostroumov et al. 2013; Tong et al. 2020), the primary focus of these studies tended to be on energy transport between the different pigment pools of B800 and B850. On the other hand, the influence of PPC structure on the intricate intraband dynamics has been much less explored.

Several factors contribute to this less extensive literature. Especially problematic is the electronic structure, which emerges from the large number of almost iso-energetic pigments making up the ring systems. The associated spectra are highly congested, with fast dephasing of optical coherences and close exciton energy-level spacing resulting in the formation of almost band-like structures. In combination with very fast relaxation dynamics, it becomes difficult to resolve the photoinduced dynamics using conventional pump-probe approaches due to their limited simultaneous time and frequency resolution.

Nevertheless, the significant structural difference between LH2 species must be expected to manifest as differences in photophysical properties, as changes to overall ring size and the details of the α/β subunit structure generically lead to changes in pigment site energies and inter-pigment coupling. Such species-dependent electronic structure and dynamics are observed in theoretical calculations (Tretiak et al. 2000) and implied by a range of spectroscopic experiments. Static experiments such as absorption spectroscopy (Kennis et al. 1997; Tong et al. 2020) and spectral hole burning (Wu et al. 1996) suggest state-energy shifts and substantial variations in homogeneous and inhomogeneous disorder. More recent work in photoinduced dynamics showed that the unique orientation of the B800 BChls in molischianum results in a split Q_x_ absorption band, with the spurious appearance of a resonance condition to a vibrational mode (Thyrhaug et al. 2018). Because of this resonance, carotenoid-to-BChl energy transfer became both ultrafast and directed to a specific sub-set of the BChl pigment pool. Similarly, the arrangement of BChls in *Allochromatium vinosum* results in a dimer-interaction split B800 band (Schröter et al. 2018). However, it is unclear to what degree such changes in electronic structure and optical spectra lead to alterations to the excited state dynamics and whether any such alteration can have consequences for light-harvesting functionality.

Here, we address this question by a direct comparison of photoinduced dynamics in two structurally and spectroscopically distinct LH2 variants extracted from the bacteria *Rhodospirillum (Phaeospirillum) molischianum* (*molischianum*) and *Rhodopseudomonas acidophila* (*acidophila*). While LH2 from both bacterial species retains the fundamental ring-like structure, in *molischianum,* LH2 has eight-fold symmetry (Fig. 1a), while in *acidophila,* it has nine-fold symmetry (Fig. 1b). Also, the details of the α/β subunits differ, with the orientation of the B800 BChls relative to those in B850 BChl being especially dissimilar. The relative Q_y_ transition dipole moment (TDM) of B800 and B850 BChls is parallel in *molischianum* and nearly perpendicular in *acidophila*. Additionally, while in *acidophila* each B800 BChl is nearly orthogonal to the B850 BChls, the B800 BChl in *molischianum* is rotated in plane by 90° and tilted by 38° (Koepke et al. 1996; Papiz et al. 2003). One might expect these differences in relative dipole moment orientation to have an influence in both intra- and interband energy transfer.

We alleviate the complications associated with the spectral congestion common to all LH2 species by employing polarization-controlled two-dimensional electronic spectroscopy (2DES). As this technique offers simultaneous optimal spectral and temporal resolution, we can extract maximal spectral information content without sacrificing the necessary time resolution. This allows us to assess both the energy-level distribution and disorder in the LH2 complexes while simultaneously following the photoinduced dynamics on timescales from tens of femtoseconds to tens of picoseconds at 80 K. In combination with full polarization control, this allows us to extract a consistent picture of the ultrafast dynamics in B800 and B850 for both investigated complexes. In doing so, we aim to assess the sensitivity of the energy relaxation cascade in LH2 to naturally occurring changes in electronic structure, and how these differences manifest.

## Results

### Static spectra: electronic structure and disorder

The low-temperature absorption spectra of LH2 from *molischianum* and *acidophila*, shown in Fig. 1c, are typical of purple bacterial light harvesters. The main spectral features are an intense absorption band near 11,750 cm^−1^ (850 nm) and a BChl-like absorption near 12,500 cm^−1^ (800 nm). These are associated with the B850 and B800 ring structures, respectively. Although the absorption spectra of the complexes are broadly similar, there are clear quantitative differences in both transition energies and lineshapes.

Energetically, the *molischianum* spectrum is shifted towards the blue by ~ 70 and ~ 120 cm^−1^ for B800 and B850, respectively, demonstrating an overall shift of the average state energies. In the B800 ring, where the pigment–pigment electronic coupling is weak, the changes to the BChl structural organization are likely predominantly associated with altered pigment site energies and, thus, an overall shift in the spectrum. Notably, though, given that the linewidths report on the energetic disorder and strength of system-environment interaction, the near-identical B800 lineshapes of *molischianum* and *acidophila* suggest that the difference in pigment organization does not induce significant differences in the width of the site-energy-distributions or -fluctuations.

The strong connection between site energies and electronic couplings inhibits definite assignments of spectral shifts for the B850 ring based on our data alone. However, it is clear that the moderate structural differences result in overall higher absorption frequencies in *molischianum*. In contrast to the B800 case, the spectra in Fig. 1c further demonstrate noticeable differences in B850 spectral lineshape between the two LH2 variants, with the *molischianum* linewidth being ~ 20% larger. This observation of higher average state energies with a broader distribution for *molischianum* agrees with earlier studies (Wu et al. 1996).

While these observations of differences in linear spectra imply that relatively minor changes in protein structure are linked with substantial differences in electronic structure and disorder, the information content in these experiments is not sufficiently detailed to draw direct links to dynamics and function. In particular, it is not trivial to link the observed changes to dynamic properties such as exciton localization, energy relaxation, and transport.

Qualitatively, however, disorder both in the form of static excitonic energy distributions and dynamic fluctuations influence the electronic- and electronic-vibrational coupling in molecular complexes, and changes in disorder can be expected to affect energy-relaxation dynamics. This is especially true if the relaxation processes rely on fine-tuned parameters such as vibrational or vibronic resonances.

Non-linear optical experiments reveal more detailed information about both dynamics and disorder. Here, we rely on 2DES to provide detailed information about the electronic state structure and disorder during the relaxation processes following photoexcitation. In particular, the diagonal and antidiagonal linewidth of 2DES signals provide convenient measures of static and dynamic disorder, respectively (Gelzinis et al. 2019).

**Fig. 2:**
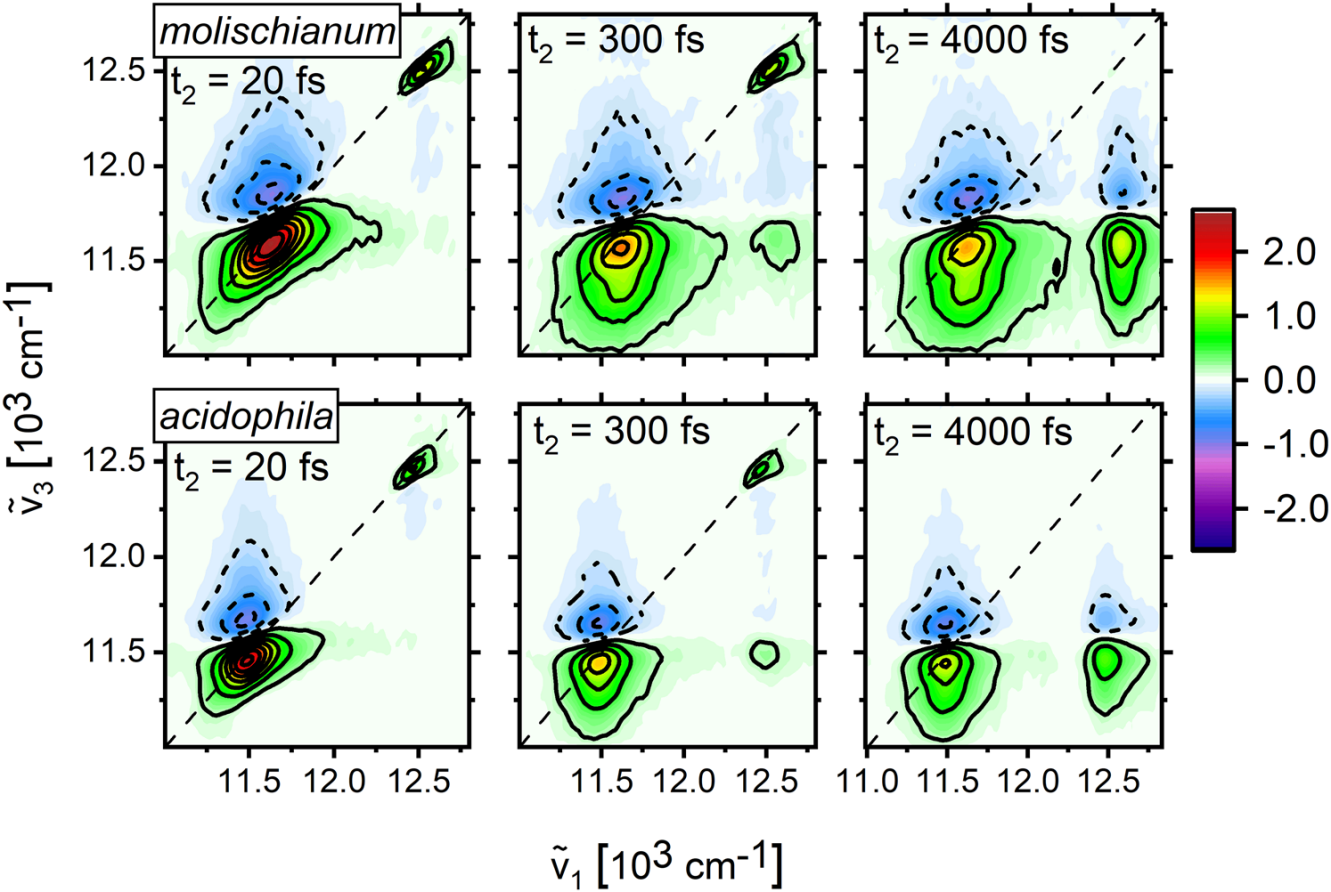
Total absorptive 2D spectra of *molischianum* (top row) and *acidophila* (bottom row) at selected population times t_2_ measured at 80 K temperature and MA polarization condition. The time evolution of the dynamics occurs on three characteristic timescales. Amplitudes normalized to the maximum at t_2_ = 20 fs

In Fig. 2, we show absorptive 2DES spectra of *molischianum* (top) and *acidophila* (bottom) at selected population times *t*_2_ covering the timescales of energy transfer in the complex. 2DES maps at later times are shown in the SI (Fig. S1). At early times, two positive features, corresponding to the B800 and B850 absorption bands, are visible along the diagonal. In accordance with the linear absorption experiments, B800 appears as a near-isolated sharp feature, while the lower-energy B850 band is significantly broader with a strongly distorted lineshape due to the overlap with a negative-amplitude excited state absorption (ESA) feature at the high-energy side. In both LH2 species, there is significant static disorder in the electronic structure. This is witnessed by the clear correlation between excitation- and detection- frequencies for both B800 and B850 features, resulting in diagonally elongated peaks at early times before the onset of energy relaxation. This correlation is quickly lost due to inter- and intra-band relaxation and interaction with the bath. For the B850 band, this process has been quantified, e.g., by following the time evolution of the nodal line slope between ESA and GSB, which decreases initially with a 60 fs time constant, followed by much slower long-time dynamics (Thyrhaug et al. 2021). It results in rounded and essentially horizontally elongated B850 features within a few hundred femtoseconds, accompanied by an overall redshift of the band.

While similar relaxation appears to take place in the B800 band, it is outcompeted by inter-band energy transfer to B850, resulting in an apparent loss of the B800-related diagonal signal before intra-band relaxation is complete (Thyrhaug et al. 2021).

**Fig. 3:**
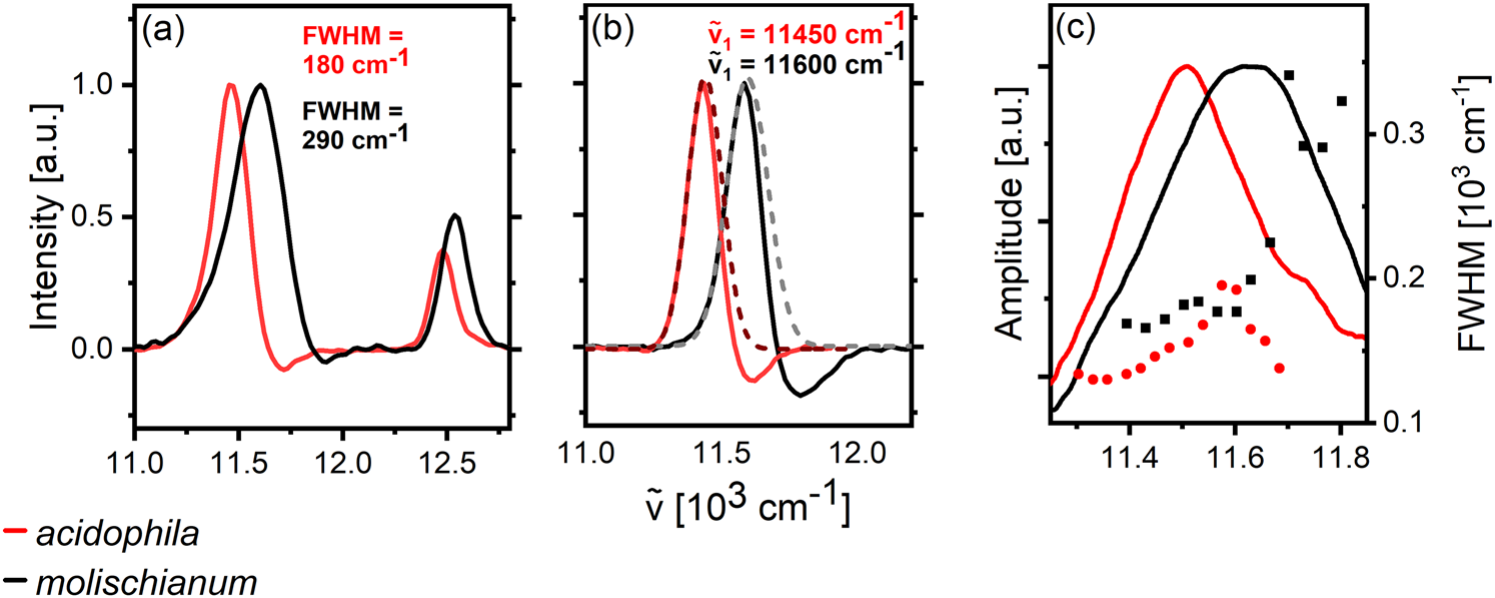
**a** Diagonal cuts through the 2DES data of molischianum and acidophila at t_2_ = 20 fs. The linewidths of both B850 peaks have been estimated by fitting them with Gaussians. **b** Representative antidiagonal cuts through the center of the B850 peak for *molischianum* and *acidophila* at t_2_ = 20 fs. The dashed lines are a Gaussian fit to the data. Fits were performed for the red edge of the peak due to overlap with ESA on the blue side and extrapolated to the entire band. The antidiagonal FWHM (dots) for fits at different excitation frequencies is shown in **c** (right y-axis) in comparison to the respective absorption spectra (lines)

Quantitative comparison of lineshapes is most conveniently done through extraction of cuts through the 2D spectra. While quantitative lineshape analysis should ideally be performed at *t*_2_ = 0 fs, we extract cuts at *t*_2_ = 20 fs as a compromise between minimizing the effects of population relaxation on the one hand and undesired pulse overlap effects on the other (Perlík et al. 2017). In Fig. 3, we show selected diagonal and anti-diagonal cuts for both complexes. The diagonal lines in Fig. 3a are slightly distorted due to the overlap with the two-exciton ESA feature, as witnessed by the minor ESA signal at the blue side of the main B850 GSB/SE feature. This minor distortion of the spectral blue edge does not introduce major inaccuracies in linewidth estimation. Assessing the anti-diagonal linewidth is more challenging as the overlap with two-exciton ESA becomes more severe. We choose to fit a Gaussian function only to the red side of the band, where this overlap is minimal, and extrapolate the fit to the whole x-axis. In Fig. 3b, we show antidiagonal cuts with their respective Gaussian fits extracted as explained above (${\widetilde{\nu }}_{1}$-values noted in the figure panel). To provide adequate estimates of the homogeneous linewidth throughout the B850 band, we here report the width of Gaussian fits to the red side of the anti-diagonal cuts.

For B800, we find that both inhomogeneous (diagonal) and homogeneous (anti-diagonal) lines are similar for the complexes (Fig. S2), indicating that the shift in site energies is not associated with significant changes in disorder. In contrast, substantial differences are apparent for the B850 band. As seen in Fig. 3a, the *molischianum* diagonal is ~ 35% broader than that of *acidophila*. The slight discrepancy between this value and that extracted from the absorption spectra (Fig. 1c) can be explained by a distortion of the diagonal peaks caused by the overlap of the ESA feature on the high-energy side. Nevertheless, it demonstrates the larger static disorder in *molischianum*, directly showing that—in contrast to B800—the structural differences in the B850 ring are not only associated with changes in state energies but also the width of the state-energy distribution (Tretiak et al. 2000).

There are notable differences in the homogeneous linewidth of B850 as well. In Fig. 3c, we show the estimated homogeneous FWHM of B850 at excitation frequencies ranging across the band. The linewidth of *molischianum* appears to be consistently larger than that of *acidophila*, in particular at higher excitation frequencies, reaching a ~ 25% difference in the respective absorption maxima and continuing to increase towards the blue. This suggests that the pigment pool in *molischianum* experiences overall larger short-timescale fluctuations than those in *acidophila*.

Overall, we thus find that the structural differences between *molischianum* and *acidophila* result in observable and non-negligible differences in electronic structure and disorder. The effects are most pronounced in the B850 ring. It would, therefore, be reasonable to expect changes in the intraband dynamics of the two LH2 complexes if the light-harvesting process relied on fine-tuned parameters such as vibrational or vibronic resonances.

### Intraband relaxation

To quantify the influence of changes to electronic structure and disorder on energy relaxation dynamics and exciton transport, we follow the photoinduced dynamics within each absorption band using polarization-controlled 2DES. This allows us to spectrally resolve ultrafast relaxation dynamics, while at the same time monitoring signal depolarization, revealing information about the spatial movement of the excitations in the system.

In agreement with the earlier work, we find that intraband relaxation in the B800 and B850 bands proceeds over a broad range of timescales. Initial dephasing of the laser-induced exciton coherence occurs within the first 50 fs (Chachisvilis et al. 1997; Vulto et al. 1999; Book et al. 2000), whereafter the population starts relaxing towards the bottom of each band. This intraband relaxation process never reaches completion in B800, as it is outcompeted by the B800→B850 energy transfer occurring on a timescale of ~ 1 ps. On the other hand, the population relaxation in the B850 band is almost complete within a few hundred fs, with only small-scale cooling taking place over the subsequent few ps (Thyrhaug et al. 2021).

Here, we predominantly focus on relaxation on hundreds of femtoseconds timescale, as the associated spectral changes and excitation-frequency dependence suggest a direct connection to intraband transport and relaxation. Such dependencies are most evident in the B800 band, where, e.g., the correlation between excitation and detection frequency is mostly preserved after the red-edge excitation, while a clear shift and broadening appear after excitation at the blue spectral edge (Fig. S3). Similarly, excitation-frequency dependent intraband relaxation over the first few hundred femtoseconds in the B850 band is obvious, albeit much more complex and requiring more careful analysis, as has been described in earlier work (Thyrhaug et al. 2021). This is due to the band-like spectral structure, which makes it inconvenient to analyze the full 2DES data directly. Instead, we extract spectral slices at all unique excitation frequencies throughout the spectrum. By this slicing of the data, we generate TA-like datasets for different pump frequencies with both high temporal and spectral resolution. Per earlier terminology, we will refer to these spectral slices as “transient hole-burning spectra” (THBS) (Thyrhaug et al. 2021).

Using the standard and well-developed scheme of global kinetic analysis (GA) (Van Stokkum et al. 2004; Snellenburg et al. 2012), we rely on the transient-absorption-like THBS to quantitatively investigate the excitation-frequency dependence of the intraband dynamics in the B800 and B850 spectral regions. This approach consists of fitting the data to a sum-of-exponentials function with global constraints on the time constants, resulting in lifetimes of individual ‘states’ or processes as well as representations of the (wavelength-dependent) amplitudes of these components in terms of Decay-associated spectra (DAS) and Evolution-associated spectra (EAS).

To characterize spatial exciton motion during energy relaxation, we supplement this global kinetic analysis with measurements of the excitation- and detection-frequency dependence of the anisotropy decay of the signal. As the anisotropy is a direct measure of the relative TDM direction of a given exciton, decay of the anisotropy can be related to a time-dependent change in the TDM direction—as has been exploited in earlier work to relate the observed energy relaxation to spatial exciton motion (Chachisvilis et al. 1997; Van Grondelle and Novoderezhkin 2006). In the case of LH2, effectively a two-dimensional system, we expect the anisotropy to decay from an initial value of 0.4 (for GSB/SE signal) to an asymptotic value of 0.1 as the TDM direction gets randomized due to the motion of the exciton around the ring.

### Dynamics in the B800 band

**Fig. 4:**
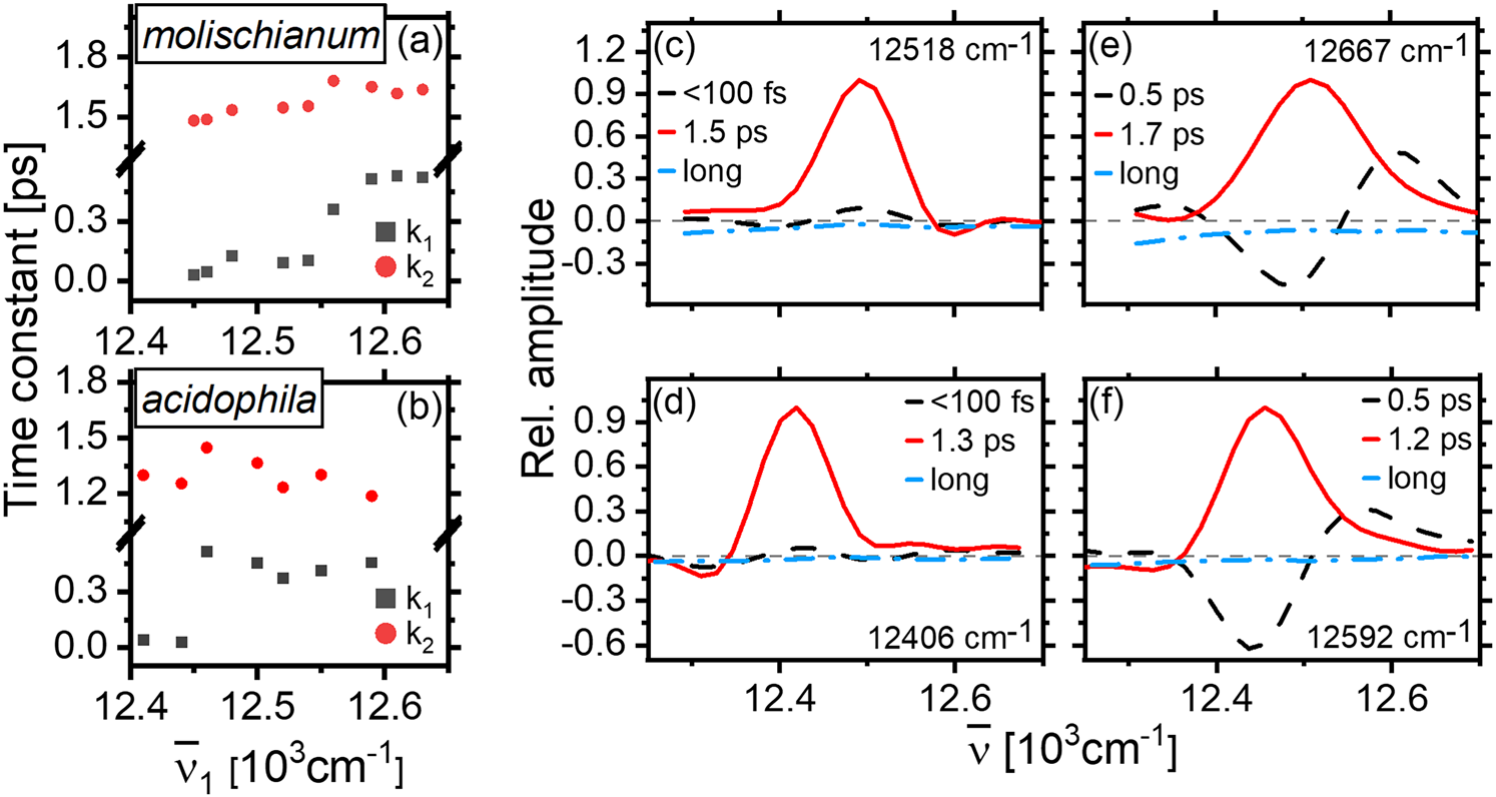
Kinetic fitting parameters for THBS in the B800 region at different excitation frequencies in *molischianum* (**a**) and *acidophila* (**b**). The DAS were extracted for each species at the red (**c**, **d**) and blue (**e**, **f**) edges of excitation

The BChl monomer-like spectra associated with the B800 ring of weakly coupled pigments suggest that intraband transport and relaxation should only be weakly dependent on changes in structure. Global analysis of THBS extracted throughout the B800 region supports this expectation. The excitation-frequency dependence of the relaxation time-constants for the two LH2 species are shown in Fig. 4a, b with selected DAS shown in panels c–f. Overall, both kinetic time constants and spectra are highly similar for the two bacterial species, and as such, we discuss them collectively here.

Three components are required to fit the THBS regardless of excitation frequency. We find sub-ps components related to the solvation and intraband relaxation, a ps component associated with the overall decay of the B800 signal, and a component of several hundred ps related to the decay of the B850 ESA.

The ps component observed at all excitation frequencies is typically assigned to an overall loss of B800 population via B800→B850 transfer (Cogdell et al. 2006). This agrees with our observation of a positive-magnitude (loss) DAS component at all excitation frequencies. Note that we observe a ~ 10% change in the value of this time-constant across the absorption band, an effect which has been assigned largely to “crosstalk” between components in the kinetic fit (Thyrhaug et al. 2021).

In the sub-ps range, the decay displays clear bimodal behavior as a function of excitation frequency. It takes on values of ~ 100 fs at the red edge while approaching 400–500 fs at the blue edge—with the inflection point occurring roughly at the B800 absorption maximum. Additionally, as displayed by the DAS in Fig. 4c–e, the component amplitude is near-negligible at the red edge, while it is substantial after blue edge excitation. The “dispersive” DAS lineshape consisting of a positive (decay) feature in the blue and a negative (grow-in) feature at the B800 center-of-mass appearing after blue-edge excitation demonstrates a clear association with downhill energy transfer. The small amplitude and less well-defined DAS lineshape appearing after red-edge excitation, on the other hand, suggests that this ~ 100 fs component is related to inertial solvation or other electronic relaxation processes in the immediate environment rather than energy transfer. The bimodal behavior observed for this component can thus be interpreted as a result of the interplay of different pathways of energy dissipation, which occur on similar timescales but whose relative importance differs depending on the wavelength of excitation.

In summary, for both bacterial species, B800 intraband energy relaxation occurs in a few hundred fs—mainly after excitation at the blue edge. The subsequent dynamics are dominated by B800→B850 energy transfer, leading to a complete loss of the B800 signal in 1–2 ps.

**Fig. 5:**
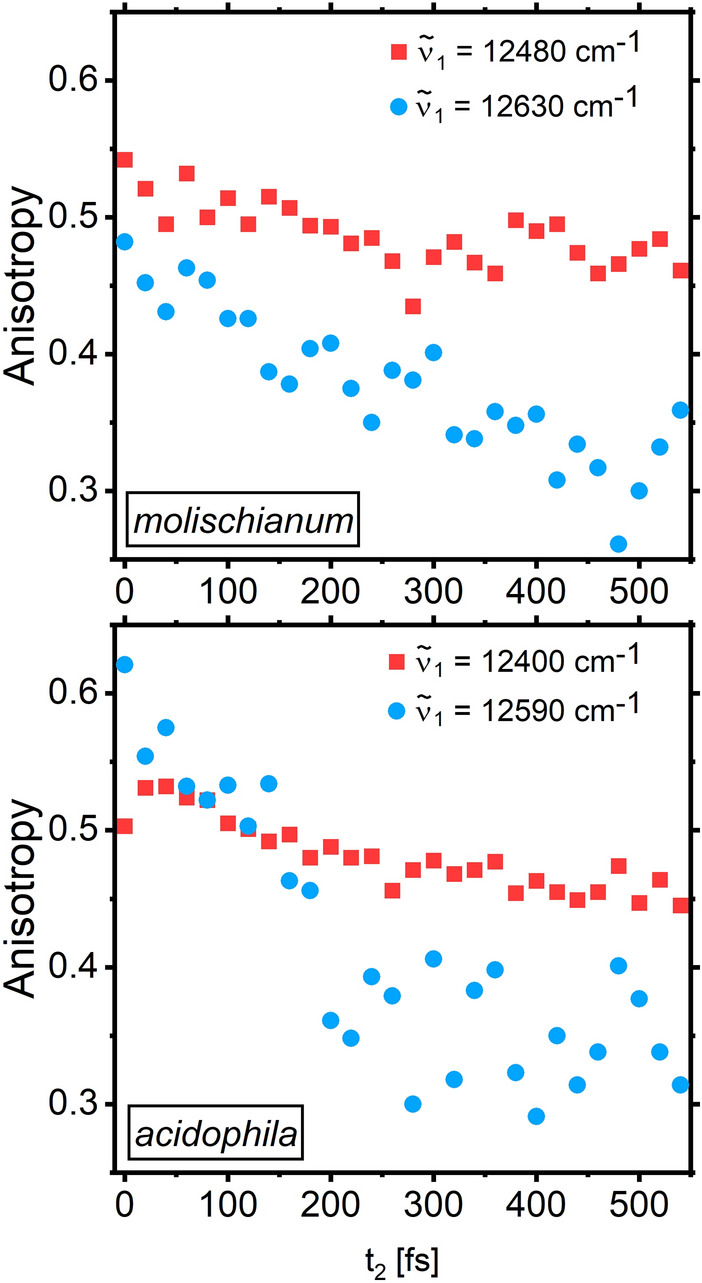
Selected anisotropy traces for the B800 band of *molischianum* (top) and *acidophila* (bottom) excited at the blue and red edge. The probe wavelength was chosen as the respective peak maximum. The anisotropy is consistently higher after red-edge excitation, indicating a lower mobility of the excitons

As a complement to the global kinetic analysis, time-resolved anisotropy supplies information on the mobility of the essentially localized excitations in B800. We show anisotropy decays after blue- and red-edge excitation in Fig. 5 for both bacterial species (complete anisotropy maps in the SI, Fig. S4). Again, the anisotropy dynamics in the two LH2 species is qualitatively identical: After blue-edge excitation the anisotropy decays rapidly, implying limited but non-negligible exciton mobility. After red-edge excitation, however, the depolarization is slow compared to the B800 lifetime. This behavior is expected for motion between weakly coupled pigments with substantial static disorder, as nearest-neighbor hopping would be strongly inhibited for low-energy excitations due to the lack of favorable spectral overlap and sufficient thermal energy to overcome the disorder. Note that the unusually large time zero anisotropy has been observed in earlier work (Thyrhaug et al. 2021), where it was assigned to overlap between GSB/SE and ESA contributions to the spectrum.

In summary, we observe no qualitative differences in the timescales of energy relaxation and excitation motion (anisotropy decay) for the two complexes, demonstrating that the substantial B800 structural differences do not significantly affect the photoinduced dynamics.

### Dynamics in the B850 band

In contrast to the B800 dynamics, intraband relaxation in the B850 band is fast and occurs in conjunction with significant spectral changes. In addition to the loss of excitation/detection frequency correlation resulting in a straightening of the GSB/ESA nodal line tilt over a few hundred fs (Thyrhaug et al. 2021), this can be observed as the appearance of a new band at the red side of the spectrum, as well as an overall red-shift of the spectral center-of-mass.

**Fig. 6:**
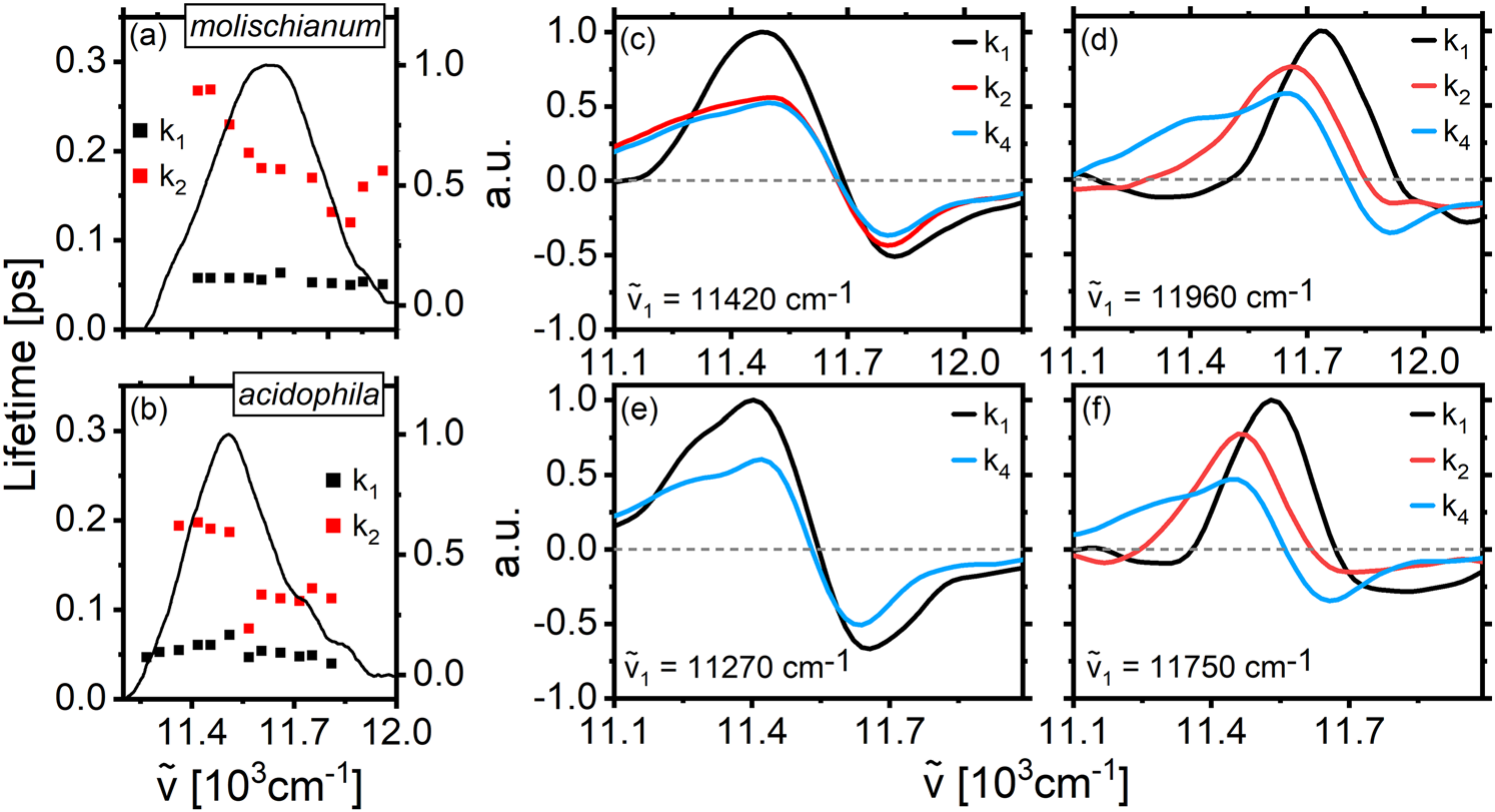
Intraband relaxation in the B850 band of *molischianum* (top) and *acidophila* (bottom). The results of the kinetic fitting are shown in panels **a** and **b** against the respective absorption spectra, while panels **c–f** show the EAS for red- and blue-edge excitation. Fitting the *molischianum* data requires four components (k_1_–k_4_) at any excitation wavelength, while three (blue edge – k_1_, k_2_, k_4_) and two (red edge – k_1_, k_4_) components are necessary to fit the *acidophila* data. A minor additional component (k_3_) in the ps range is needed for the *molischianum* data. It appears unrelated to electronic energy relaxation and has been omitted from the figure, but the associated lifetimes and EAS can be found in the SI (Fig. S5). The fourth component, k_4_, has a long lifetime associated with ground-state recovery and is not shown in **a/b**, but the corresponding DAS are shown in blue in panels **c–f**

Our analysis of the B850 relaxation dynamics is analogous to our treatment of the B800 dynamics, relying on global analysis of individual THBS. We find that both LH2 species show multi-exponential dynamics regardless of excitation frequency, with the dominating timescales being largely in the sub-ps range. We show these sub-ps time constants as a function of excitation frequency for both LH2 species in Fig. 6a, b.

Due to the complex dynamics and heavily congested spectra, the DAS representation of the decay component amplitudes becomes cumbersome to analyze (Marciniak and Lochbrunner 2014). Therefore, we instead use the EAS representation, where the underlying kinetic model is strictly sequential state-to-state transfer. As the B850 excitonic states are not individually spectrally resolvable, the EAS should not be interpreted as eigenstate spectra but rather as spectra of “effective” states populated during the evolution. The EAS including the kinetic components k_1_, k_2_ and k_4_ at selected excitation frequencies are shown in panels c-f of Fig. 6. Component k_3_ is not shown as it appears unrelated to electronic energy relaxation, but the associated lifetimes and EAS can be found in the SI (Fig. S5).

In both species, we initially observe large spectral changes taking place on a timescale of ~ 50 fs (k_1_), essentially independent of excitation frequency. The corresponding EAS in Fig. 6c–f reveals this component to be of large amplitude and sharply peaked in the range of the absorption center-of-mass. Following earlier work, we assign this component to dynamic localization of the initially excited Bloch-wave-like state (Book et al. 2000; Dahlbom et al. 2001; Thyrhaug et al. 2021).

Following dynamic localization, we observe a component k_2_ that is clearly associated with intraband energy relaxation. As seen in Fig. 6a, b, this component demonstrates strong excitation-frequency dependence in both lifetime and spectral shape, with generally faster relaxation after blue edge excitation. Towards the red edge, the dynamics slow down. Notably, both LH2 species show qualitatively identical behavior both in terms of time-constant magnitude and its change across the absorption band.

After excitation at the very red edge, the EAS amplitude of k_2_ (red line in Fig. 6c, d, f) decreases substantially—ultimately disappearing entirely in the case of *acidophila* (Fig. 6e). This change in k_2_ amplitude across the band is mirrored in its EAS spectral shape: excitation at the blue edge (Fig. 6d, f) results in relatively sharp and narrow lineshapes that are highly dissimilar to the EAS of the fully relaxed excited state (k_4_). After red-edge excitation, on the other hand, the k_2_ EAS is almost identical to the fully relaxed long-time spectrum (k_4_, Fig. 6c). As such, this component appears to be the main indicator of downhill energy transfer after high-energy excitation. While still present at the spectral red edge, the small amplitude and only subtle spectral changes suggest that excitation here is essentially directly into the lowest energy states of the system.

After completed excited-state relaxation, both species show an excitation-wavelength-independent component (k_4_) of several hundred picoseconds, corresponding to the recovery of the ground state. Also, these spectra are highly similar between the LH2 species, indicating that there is no substantial difference in their relaxed excited states.

We note that a fit to *molischianum* data requires an additional minor component (k_3_) in the ps range, which appears unrelated to electronic energy relaxation. Due to its small amplitude and very subtle effect on the spectra, we assign this to a small-scale reorganization process. The inclusion of this minor component is not necessary to achieve a satisfactory fit of the *acidophila* data.

**Fig. 7:**
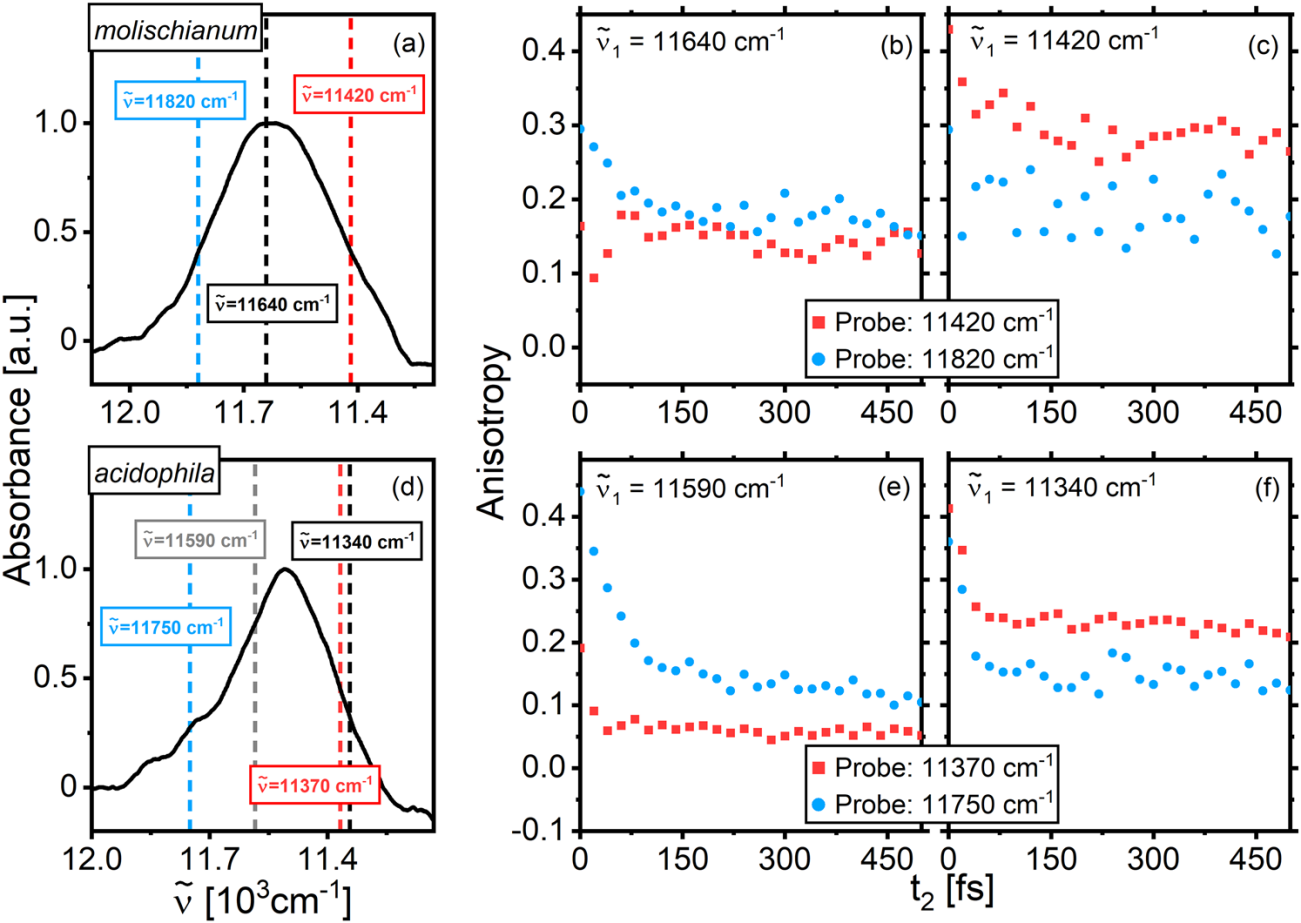
Single-point anisotropy decay traces for* molischianum* and *acidophila* at blue (**b, e**)—and red-edge (**c, f**) excitation. The excitation and detection energies are shown against the absorption spectra in **a, b**. For red-edge excitation and detection, the depolarization is noticeably slower: the anisotropy only decays to a value of r = 0.25‒0.3, while it reaches values of 0.1–0.2 for all other excitation/detection combinations

To connect the global analysis to spatial exciton motion in B850, we show anisotropy decays extracted at selected detection frequencies after blue- and red-edge excitation in Fig. 7. The full anisotropy decay maps can be found in Fig. S6.

For both species, we find that the anisotropy decays biexponentially, with one very fast component (20–50 fs) and one in the range of several ps. Although the shorter component is likely partly related to the initial dephasing dynamics, its value is close to that of the pulse auto-correlation, inhibiting detailed quantitative analysis. It has, however, been shown that (at least in porphyrin dimers, trimers, and hexamers) an anisotropy decay of < 50 fs is consistent with a nearest-neighbor hopping model with a transfer time of < 100 fs (Cho et al. 2003). Interestingly, after excitation at the red side of the absorption maximum, little depolarization is observed after the initial 50 fs, although the GA indicates that energy relaxation continues over several 100 fs (cf. Figure 6, in particular component k_2_). This suggests that the energy relaxation process occurring at this timescale is at least in part spatially local, as the lack of depolarization implies excitons that are essentially stationary.

In summary, the combined analysis of energy transfer dynamics by GA and polarized data shows that LH2 complexes from *molischianum* and *acidophila* are functionally identical. In both the B800 and B850 rings, we observe ultrafast exciton localization, followed by depolarization and intraband energy relaxation when the excitation energy is sufficiently high. This is followed by B800→B850 transfer in a few ps, prior to small-scale cooling processes. For both species, the excited-state dynamics involve both spatial motion and local energy-relaxation. While this behavior is complex and not understood in detail, we note that we observe no qualitative differences between the two LH2 complexes despite large structural differences in the B800 region and clear differences in the electronic structure and disorder, particularly in the B850 region.

## Conclusions

Our results for *acidophila* agree with those of earlier work on intraband relaxation in this system (Thyrhaug et al. 2021). The comparison with *molischianum* reveals that the light-harvesting photophysics is very similar for the two complexes despite significant structural differences, particularly in the arrangement of the B800 pigments, and despite large changes in electronic structure and disorder as shown by lineshape analysis. This suggests that the LH2 light harvesting functionality does not depend heavily on fine-tuned parameters such as vibrational or vibronic resonances and is robust against electronic structure detuning.

Further, the existence of a local energy relaxation process that does not involve exciton motion several 100 fs after the initial dephasing is evident in both bacterial species. This process is most pronounced after excitation in the red edge of the absorption band. Similar behavior has been observed in hole-burning experiments, where this exciton self-trapping was assigned to a polaron formation (Polívka et al. 2000; Timpmann et al. 2001). Interestingly, this component is very similar in both complexes despite their differences in structure and disorder.

While the similarities in dynamics between the complexes are striking, we emphasize that relating the dynamics of isolated pigment-protein complexes to the in vivo functionality of the photosynthetic system is not straightforward. Our experiments reveal how structural differences influence exciton relaxation dynamics within the LH2 complexes. To what degree dynamics at this early stage of the photosynthetic process are relevant for the subsequent (and much slower) processes in the photosynthetic apparatus remains an interesting, but open question.

In summary, our results show that changes in TDM orientation, site energies, disorder, and electronic structure have little influence on the light-harvesting photophysics of LH2. By quantitatively comparing inter- and intraband relaxation in complexes from *molischianum* and *acidophila*, which exhibit clear structural differences, we conclude that the light harvesting functionality of LH2 is a robust process that is insensitive to fine-tuning of parameters but rather proceeds on similar timescales and with similar trends across systems.

## Experimental methods

### Technical details

The instrument used for 2DES experiments is described in detail elsewhere (Augulis et al. 2011). In summary, a 1030 nm Yb:KGW laser (Pharos, Light Conversion ltd.) was used to pump a lab-built noncollinear optical amplifier, the pulses of which were compressed by a combination of chirped mirrors and a fused silica prism compressor. The output was approximately 12 fs pulses with a spectrum centered at 830 nm. To avoid artifacts related to exciton-exciton annihilation, the pulse energy was kept at 600 pJ. The beams were focused into a ~ 160 µm diameter spot in the sample. All experiments were done at a 20 kHz repetition rate. The coherence time was scanned from ‒230 to 500 fs with 2 fs steps, resulting in a spectral resolution of 33 cm^−1^ on the ṽ_1_ axis; the resolution on the ṽ_3_ axis was 55 cm^−1^. The linear polarizations of the pulses were independently controlled by a combination of a quarter-wave plate and linear wire-grid polarizers in each beam. To avoid depolarization artifacts, we measured population dynamics at magic angle conditions (< 54.7, 54.7, 0, 0 >), while anisotropies were calculated as usual as a combination of the < 0, 0, 0, 0 > and < 90, 90, 0, 0 > sequences of linearly polarized pulses.

### Sample preparation and experiment

LH2 from *acidophila* and *molischianum* were extracted, isolated, and purified according to earlier detailed procedures (Gardiner et al. 2018; Thyrhaug et al. 2018). The purified samples were stored at ‒80/40  C in a buffer until immediately before use. Before the experiment, the LH2 stock solution was diluted in TRIS–HCl buffer containing LDAO, glucose, glucose oxidase, and catalase. The resulting buffer solution was mixed to a 1:2 ratio with glycerol to reach an absorbance of ~ 0.4 in a 200 µm optical path cell, whereafter the sample was immediately inserted in a liquid nitrogen flow cryostat (Oxford Instruments) and kept at 80 K throughout the measurement.

### Data analysis and visualization

The crystal structures for both bacterial LH2 complexes were retrieved from the RCSB protein data bank (experimental PDB structure 1NKZ for *Rps. acidophila* from J Mol Biol **326**: 1523–1538, 2003 and experimental PDB structure 1LGH for *R. molischianum* from Structure **4**: 581–597, 1996) and visualized with Mol*. The global analysis was done using the open-source software Glotaran (Snellenburg et al. 2012).

### Supplementary Information

Below is the link to the electronic supplementary material.

Supplementary file1 (DOCX 2046 KB)

## Data Availability

The data that support the findings of this study are available from the corresponding author upon reasonable request.
