# Supplementary material for: Light harvesting in purple bacteria does not rely on resonance fine-tuning in peripheral antenna complexes
Source: Photosynth Res. 2024 Jun 21;161(3):191–201. doi: 10.1007/s11120-024-01107-4 (PMC11324704; doi:10.1007/s11120-024-01107-4)
Supplement: Supplementary file 1 — Supplementary file1 (DOCX 2046 KB) [file 11120_2024_1107_MOESM1_ESM.docx]

**Light harvesting in purple bacteria does not rely on resonance fine-tuning in peripheral antenna complexes**

Erika Keil, Heiko Lokstein, Richard Cogdell, Jürgen Hauer, Donatas Zigmantas, and Erling Thyrhaug

**Supporting Information**

**

**

**S1:** Total absorptive 2D spectra of *molischianum* (top) and *acidophila* (bottom) at t_2_ = 10 ps measured at 80 K temperature and MA configuration. Spectral dynamics at this and longer timescales are only an overall loss of signal amplitude due to ground-state recovery.

**S2:** Diagonals (left) and representative antidiagonals (right) in the B800 region of LH2 from acidophila and molischianum at t_2_ = 20 fs (antidiagonal peaks were shifted along X to overlap). The FWHMs were estimated by fitting Gaussians to the peaks and are similar for the two bacterial species.

**S3:** Representative 2DES spectra in the B800 region from molischianum (a, b) and acidophila (c, d) show the evolution of the B800 band along the population time t2. Excitation frequency dependence on the intraband relaxation is clearly visible: while the excitation and detection frequencies are strongly correlated at early times, the peak shifts and broadens after some hundred fs. This effect is strongest at the blue edge of the peak.

**S4:** Full anisotropy maps for the B800 band of R. molischianum (top) and Rps. acidophila (bottom) excited at the band's red (left) and blue (right) edge and overlaid with the THBS at the same excitation frequency.


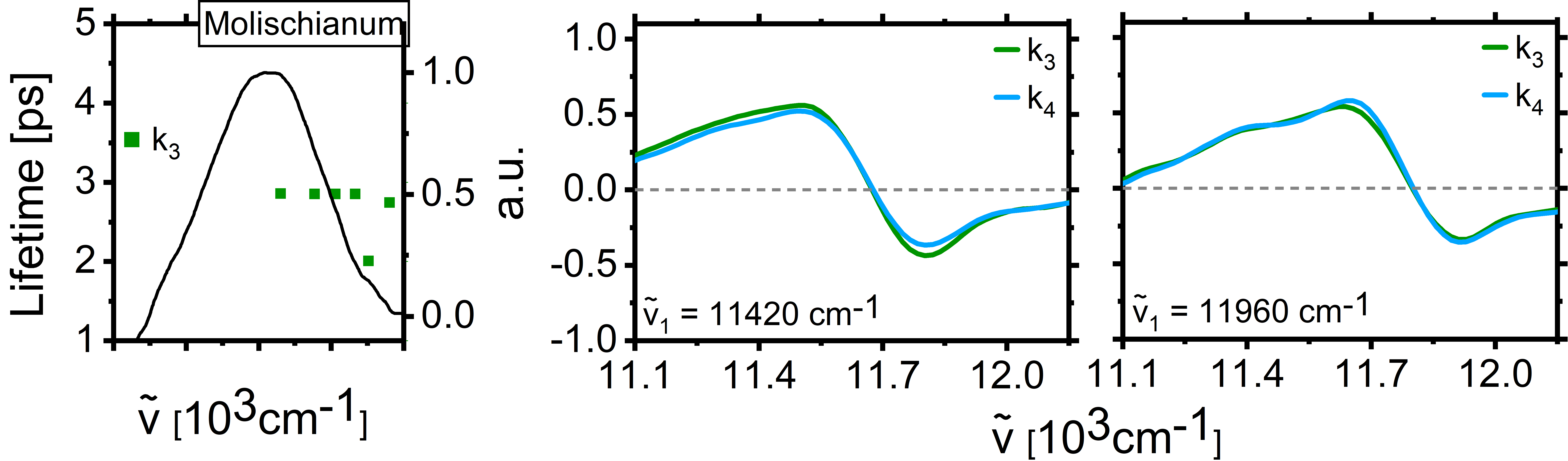


**S5:** Kinetic component k_3_ obtained from the global analysis of the B850 band of molischianum (top). The results of the kinetic fitting are shown in the left panel against the absorption spectrum, while the center and right panels show the EAS for red- and blue-edge excitation. The additional component (k_3_) needed for the molischianum data is in the ps range and appears unrelated to electronic energy relaxation, as its EAS are almost identical to those of the relaxed state (k_4_, shown in light blue for comparison).

**S6:** Full anisotropy maps for the B850 band of R. molischianum (top) and Rps. acidophila (bottom) excited at the band's red (left) and blue (right) edge and overlaid with the THBS at the same excitation frequency.
